# Impact of housing on the survival of persons with AIDS

**DOI:** 10.1186/1471-2458-9-220

**Published:** 2009-07-07

**Authors:** Sandra K Schwarcz, Ling C Hsu, Eric Vittinghoff, Annie Vu, Joshua D Bamberger, Mitchell H Katz

**Affiliations:** 1 San Francisco Department of Public Health, San Francisco, USA; 2 Department of Epidemiology and Biostatistics, University of California, San Francisco, San Francisco, USA

## Abstract

**Background:**

Homeless persons with HIV/AIDS have greater morbidity and mortality, more hospitalizations, less use of antiretroviral therapy, and worse medication adherence than HIV-infected persons who are stably housed. We examined the effect of homelessness on the mortality of persons with AIDS and measured the effect of supportive housing on AIDS survival.

**Methods:**

The San Francisco AIDS registry was used to identify homeless and housed persons who were diagnosed with AIDS between 1996 and 2006. The registry was computer-matched with a housing database of homeless persons who received housing after their AIDS diagnosis. The Kaplan-Meier product limit method was used to compare survival between persons who were homeless at AIDS diagnosis and those who were housed. Proportional hazards models were used to estimate the independent effects of homelessness and supportive housing on survival after AIDS diagnosis.

**Results:**

Of the 6,558 AIDS cases, 9.8% were homeless at diagnosis. Sixty-seven percent of the persons who were homeless survived five years compared with 81% of those who were housed (p < 0.0001). Homelessness increased the risk of death (adjusted relative hazard [RH] 1.20; 95% confidence limits [CL] 1.03, 1.41). Homeless persons with AIDS who obtained supportive housing had a lower risk of death than those who did not (adjusted RH 0.20; 95% CL 0.05, 0.81).

**Conclusion:**

Supportive housing ameliorates the negative effect of homelessness on survival with AIDS.

## Background

Homelessness is associated with excess morbidity and mortality [1-4]. Homeless persons suffer from high rates of substance abuse [5-7], mental illness [5,7-9], tuberculosis [10,11], infectious hepatitis [6,12-15], and insufficient health care [14,15].

Among HIV-infected persons, unstable housing has been associated with fewer ambulatory care visits [16], greater reliance on emergency departments [16-19], frequent or longer hospitalizations [16,17,19,20], and decreased use of antiretroviral therapy and prophylaxis against opportunistic illnesses [17,18,20,21]. Among homeless persons who have been prescribed antiretroviral therapy, adherence is suboptimal [17,22].

Mortality among HIV-infected persons with unstable housing has not been well-studied. Two studies found no effect of homelessness on AIDS survival; however, one of the studies was conducted before the availability of effective antiretroviral therapy [23] and the other did not include HIV-infected persons with stable housing as a comparison group [6]. More recently, a clinic-based, case-control study of HIV patients [24] and an analysis of data from two cohort studies of HIV infection and alcohol use found that homelessness independently predicted mortality [25]. To date, however, no studies have examined the effect of homelessness on AIDS survival in a population-based sample or the effect of providing supportive housing on survival in the era of effective antiretroviral therapy.

## Methods

### Study population

All adult and adolescent (aged ≥13 years) San Francisco residents who were diagnosed with AIDS from January 1, 1996 through December 31, 2006 and reported to the San Francisco Department of Public Health (SFDPH) by November 30, 2007 were included in the study. The AIDS surveillance system is evaluated annually and consistently found to be over 95% complete [26].

More than 90% of AIDS cases undergo a complete medical chart review at the time of report with records re-reviewed and updated every 18–24 months. Data collected include demographic and risk information, insurance status, AIDS-defining illnesses, results of HIV, CD4, and viral load tests, date of initiation and type of antiretroviral therapies, and of prophylaxis against *Pneumocystis jirovecii *pneumonia (PCP) and *Mycobacterium avium *complex (MAC).

Housing status is collected at diagnosis. Cases were considered to be homeless if the medical record noted that the patient was homeless or if the address recorded was a known homeless shelter, a health care clinic, or a free postal address not connected to a residence (e.g., general delivery). Persons with missing addresses in the medical record were considered to be housed.

Documentation of deaths was obtained through weekly review of local death certificates, reports from other health departments, and annual matches with the National Death Index, which includes deaths through 2005. Underlying and contributory causes of death were coded according to the International Classification of Diseases – 9^th ^and 10^th ^revisions.

### Comparison of survival among homeless and housed persons

Differences in the characteristics of homeless and housed persons were assessed using the chi square test for differences in proportions and the *t *test for differences in means. The Kaplan-Meier method was used to estimate the distribution of the time from AIDS diagnosis to death among homeless and housed persons; cases not known to have died were censored at the more recent of either the date of their last follow-up or December 31, 2005. The log-rank test was used to assess differences in survival. As with previous studies of AIDS survival, we used all-cause mortality [27-29].

A Cox proportional hazards model was used to estimate the independent associations of housing status with mortality. For the multivariable analysis, we included demographic and risk characteristics, insurance status at diagnosis, the AIDS-defining condition (low CD4 count versus an opportunistic illness), the CD4 count at diagnosis, use of antiretroviral therapy (as a time-dependent variable), and prophylaxis against PCP and MAC.

To assess the proportionality assumption, we checked for interaction between each risk factor and time since AIDS diagnosis; for highly active antiretroviral therapy, a time-dependent covariate, we assessed interaction with time since start of treatment. Because the risk associated with homelessness increased during the study period (p < 0.05), we calculated the estimated relative hazard (RH) at one, three, and five years after diagnosis. Results did not suggest qualitative changes from harm to protection or from protection to harm; therefore, we also calculated a summary RH for this predictor, averaged over the entire follow-up period.

To examine in more detail the factors related to high mortality in homeless people, we compared the five most frequent contributory causes of deaths among homeless and housed AIDS cases, as obtained from the National Death Index.

### Effect of obtaining housing on AIDS survival

The SFDPH provides supportive housing services to homeless persons who have the most severe or greatest number of chronic medical or psychiatric needs, or both, through the Direct Access to Housing (DAH) program. The program houses people directly from shelters, street living situations, or institutions. Residents must pay rent on a sliding scale of 30% to 50% of their income; those who are ineligible for public entitlements are housed for free. All DAH sites have dedicated case managers and provide medical services that range from an on-site, full-time nurse and part-time, mid-level clinician (e.g. nurse practitioner) to obtaining care at designated health care facilities located near the DAH residences. Supportive housing services were initiated in 1999, although the DAH program database includes move-in dates as early as 1992. The database contains the names, dates of birth, and move-in dates of persons who are housed by the program.

To identify homeless AIDS cases who obtained DAH housing, we computer matched the AIDS case registry to the DAH database using the patient name and date of birth as the matching variables. Cases who did not match using these criteria were matched using the soundex code (an alpha numeric code that allows for minor differences in the spelling of names) of the last name, first initial of the first name, and date of birth. All matches were manually reviewed. When available, matched cases were further compared using sex and race. To be classified as a homeless AIDS patient who obtained DAH housing, the move-in date must have been after diagnosis. By matching all AIDS cases to the DAH database, we identified persons who were not originally reported as homeless but received supportive housing. Because the DAH program offers housing only to persons who have been identified by their case manager as homeless, AIDS cases who were housed through DAH were included in the analysis of the impact of housing on survival and considered to have been homeless at the time of diagnosis as long as housing was obtained after the AIDS diagnosis. Such individuals were likely misclassified as housed at AIDS diagnosis or they may have become homeless sometime later.

Differences in the characteristics of homeless persons who received housing and those who did not were assessed using the chi square test for categorical variables and the *t *test for continuous variables. A multivariable proportional hazards model was performed to estimate the independent association of supportive housing with mortality. Obtaining public housing was included as a time-dependent variable to avoid creating a survival bias among those surviving long enough to obtain housing. Included in the multivariable model were demographic and risk characteristics, the AIDS-defining diagnosis (low CD4 count or opportunistic illness), the CD4 count at diagnosis, use of antiretroviral therapy (as a time-dependent variable), and prophylaxis against PCP and MAC. Because there were so few cases with private insurance, this variable was excluded from the model. The proportionality assumption was assessed using the methods previously described. No statistically significant violations were found.

### Estimating the cost per year of life saved of providing housing

To estimate the increase in average survival time over the first five years in supportive housing, we matched each homeless AIDS case who obtained housing to one control who did not. The cases and controls were matched by date of AIDS diagnosis (plus or minus six months), CD4 count at diagnosis (≤100, 101–200, >200), age at diagnosis (<40 years to ≥40 years), and whether they had injected drugs. Some cases matched with more than one control; in this situation, we selected the control whose date of diagnosis and CD4 cell count most closely matched. To avoid both a survival bias and a potential bias from controls being denied housing because of imminent death, controls were required to survive at least the same number of months from date of diagnosis as the case at the time that the case obtained housing and at least three months beyond the date that the case obtained housing. Follow-up time for cases and controls began the date the case obtained housing.

Using the matched sample, we estimated the increase in mean survival time over the first five years after receipt of housing for cases and their matched controls by calculating the difference in areas under the Kaplan-Meier survival curves [30]. The survival time for cases and controls still alive and in follow-up five years or more after receiving supportive housing was considered to be five years. Housing costs were limited to those incurred by the health department: the lease, property management, and support services. We estimated the cost of housing as the restricted mean survival time in the housed group multiplied by the average monthly cost of housing. The cost per year of life saved then was estimated as the annualized average cost of housing divided by the increase in the restricted mean survival gain associated with housing. We used bootstrap methods to compute 95% bias-corrected percentile confidence limits (CL) for restricted mean survival, survival gain from housing, and the cost per year of life saved [31].

**Figure 1:**
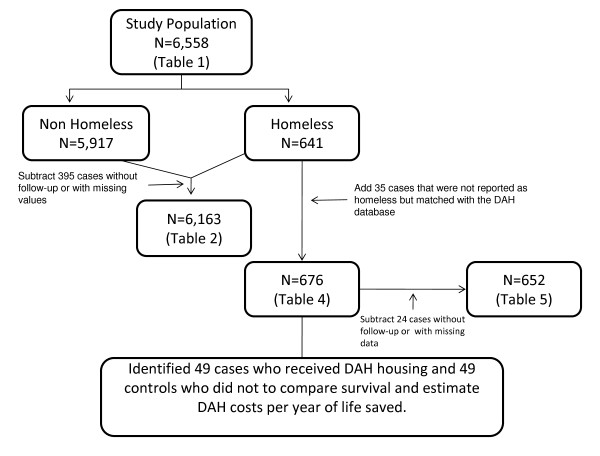
**This figure displays the samples used to construct each table.** This figure shows where each of the samples used in the analyses that produced the results displayed in each of the tables as well as the reason that data were added or deleted.

Figure 1 displays the derivation and composition of the samples used in each of the analyses. In the proportional hazards models, records with missing values were excluded. Analyses were done using SAS (SAS Institute, Cary, North Carolina) and Stata software (StataCorp, College Station, Texas). The study protocol was approved by the Institutional Review Board of the University of California, San Francisco.

## Results

### Comparison of persons by housing status at the time of AIDS diagnosis

Of the 6,558 AIDS cases included in this analysis, 641 (9.8%) were homeless at diagnosis. Homeless cases were more likely to be women or transgender, less than 30 years old, African American, and injection drug users, to have public health insurance or to be uninsured, and to be less likely to be aged 50 years or older, or to be receiving antiretroviral therapy than were the housed cases (Table 1).

**Table 1:** Characteristics of persons diagnosed with AIDS in San Francisco, 1996–2006, by housing status at diagnosis (N = 6558)

**Characteristics**	**Homeless**		**Non-Homeless**		
	**(N = 641)**		**(N = 5917)**		

	N	%	N	%	P value*

**Gender**					<0.0001

Male	497	77.54	5363	90.64	

Female	94	14.66	399	6.74	

Transgender	50	7.80	155	2.62	

					

**Age at diagnosis (years)**					0.0003

13–29	83	12.95	539	9.11	

30–39	255	39.78	2361	39.90	

40–49	229	35.73	2032	34.34	

50+	74	11.54	985	16.65	

					

**Race/ethnicity**					<0.0001

White	260	40.56	3646	61.62	

African American	252	39.31	963	16.28	

Latino	106	16.54	944	15.95	

Asian/Pacific Islander/Native American/Other	23	3.59	364	6.15	

					

**Risk group**					<0.0001

Men who have sex with men	135	21.06	4098	69.26	

Injection drug user	257	40.09	634	10.71	

Men who have sex with men and inject drugs	213	33.23	881	14.89	

Heterosexual/Other	36	5.62	304	5.14	

					

**Insurance status**					<0.0001

Public	212	33.07	1205	20.37	

Private	7	1.09	2628	44.41	

None	408	63.65	1906	32.21	

Unknown	14	2.18	178	3.01	

					

**Initial AIDS diagnosis**					0.3518

CD4 count <200 cells/mm^3^	503	78.47	4735	80.02	

Opportunistic illness	138	21.53	1182	19.98	

					

**CD4 count at diagnosis (mean cells/mm^3^)^†^**		172		184	0.0346

					

**Ever received highly active antiretroviral therapy**					<0.0001

Yes	454	70.83	4870	82.31	

No	187	29.17	1047	17.69	

					

**Ever received prophylaxis against *Pneumocystis jirovecii *pneumonia**					

Yes	417	65.05	3384	57.19	0.0001

No	224	34.95	2533	42.81	

					

**Ever received prophylaxis against *Mycobacterium avium *complex**					

Yes	173	26.99	1099	18.57	<0.0001

No	468	73.01	4818	81.43	

* Chi square test for differences in proportions and t test for differences in means.

† Excludes 205 cases that were missing CD4 results.

**Figure 2:**
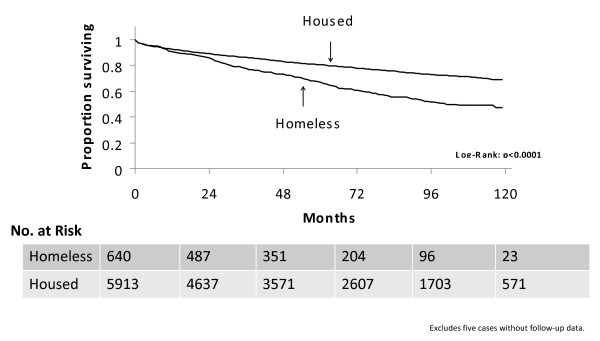
**Kaplan-Meier survival curves for San Francisco AIDS cases diagnosed between 1996–2006, by housing status at diagnosis**. The data displayed represent the proportion of persons living (in months) following the date of their AIDS diagnosis, stratified by housing status at the time of AIDS diagnosis (housed, or homeless). x axis: time (months)y axis: proportion (percent) surviving

Survival was significantly worse for homeless persons. Sixty-seven percent of persons who were homeless at the time of AIDS diagnosis survived five years compared with 81% of housed persons (p < 0.0001, Figure 2).

After adjustment for potential confounders, homelessness was significantly associated with increased mortality (RH 1.20; 95% CL 1.03, 1.41; Table 2). The analysis of the changes in the RH over time showed that the adjusted increase in the risk of mortality associated with homelessness increased from 1.00 (95% CL 0.82, 1.22) at one year, to 1.22 (95% CL 1.04, 1.42) at three years, and to 1.48 (95% CL 1.22, 1.79) at five years.

**Table 2:** Multivariable predictors of mortality among persons diagnosed with AIDS in San Francisco, 1996–2006 (N = 6163)*

**Characteristics**	**Adjusted Relative Hazard**	**95% Confidence Limits**
		

**Homeless at AIDS diagnosis**		

No	Referent	

Yes	1.20	1.03, 1.41

		

**Gender**		

Male	Referent	

Female	0.80	0.65, 0.98

Transgender	1.14	0.87, 1.51

		

**Age at diagnosis (years)**		

<40	Referent	

≥ 40	1.67	1.50, 1.86

		

**Race/ethnicity**		

White	Referent	

African American	0.96	0.83, 1.10

Latino	0.83	0.70, 0.98

Asian/Pacific Islander/Native American/Other	0.77	0.59, 0.99

		

**Risk group**		

Men who have sex with men	Referent	

Injection drug user	1.88	1.59, 2.23

Men who have sex with men and inject drugs	1.52	1.32, 1.76

Heterosexual/Other	1.48	1.14, 1.93

		

**Insurance status**		

Private	Referent	

Public	2.12	1.82, 2.48

None	1.60	1.39, 1.84

		

**Initial AIDS diagnosis**		

CD4 count < 200 cells/mm^3^	Referent	

Opportunistic illness	1.41	1.24, 1.60

		

**CD4 count at diagnosis^†^**	0.90	0.88, 0.92

		

**Ever received highly active antiretroviral therapy**		

No	Referent	

Yes	0.82	0.72, 0.93

		

**Ever received prophylaxis against *Pneumocystis jirovecii *pneumonia**		

No	Referent	

Yes	0.89	0.80, 1.00

		

**Ever received prophylaxis against *Mycobacterium avium *complex**		

No	Referent	

Yes	0.94	0.83, 1.07

* Excludes cases without follow-up or with missing values.

† Included as a continuous variable using incremental categories of 50 cells.

Other significant predictors of mortality were older age at diagnosis (RH 1.67; 95% CL 1.50, 1.86), injection drug use among persons other than men who have sex with men (MSM) (RH 1.88; 95% CL 1.59, 2.23), injection drug use among MSM (RH 1.52; 95% CL 1.32, 1.76), heterosexual contact (RH 1.48; 95% CL 1.14, 1.93), having public or no health insurance at diagnosis (RH 2.12; 95% CL 1.82, 2.48 and RH 1.60; 95% CL 1.39, 1.84, respectively), and having an opportunistic illness as the AIDS defining diagnosis (RH 1.41; 95% CL 1.24, 1.60). Use of antiretroviral therapy, prophylaxis against PCP, and higher CD4 counts at diagnosis were all associated with decreased risk of death (Table 2).

Although HIV/AIDS was the most frequent cause of death for all homeless persons with AIDS, the proportion of homeless persons who died from HIV/AIDS was significantly lower than for housed cases (Table 3). Causes of death that are associated with substance use (e.g., hepatitis, other causes of liver disease, septicemia) and mental illness occurred more frequently among the homeless. In contrast, chronic diseases were the most frequent non-HIV/AIDS-related causes of death among the housed cases.

**Table 3:** Multiple causes of death among persons diagnosed with AIDS between 1996 and 2005, by homeless status at diagnosis

	**Homeless**	**Housed**
**Cause of death**	**N (%)**	**N (%)**

		

HIV/AIDS	156 (78%)	905 (83%)

Hepatitis	41 (21%)	130 (12%)

Liver disease	39 (20%)	160 (15%)

Septicemia	35 (18%)	121 (11%)

Mental illness	33 (17%)	52 (5%)

Heart disease	29 (15%)	201 (18%)

Pneumonia (non-AIDS-related)	27 (14%)	172 (16%)

Non-AIDS cancer	12 (6%)	139 (13%)

P = 0.02 using the chi square test for differences between HIV/AIDS-related deaths and non-HIV/AIDS-related deaths.

### Impact of obtaining housing on AIDS survival

Seventy AIDS cases matched with the DAH database and had move-in dates that were later than the AIDS diagnosis date, 35 of these cases were listed as homeless in the AIDS registry at the time of diagnosis and the other 35 were added after database matching. The result was a total of 676 homeless persons for this analysis (70 housed and 606 non-housed). The characteristics of persons who received housing and those who did not were similar except those who received housing were older (Table 4).

**Table 4:** Characteristics of homeless persons with AIDS who did and did not receive health department supportive housing, in San Francisco, 1996–2006 (N = 676)*

**Characteristics**	**Received Supportive Housing**				
	**Yes**		**No**		

	**(N = 70)**		**(N = 606)**		

	N	%	N	%	P value^†^

**Gender**					0.1448

Male	48	68.57	472	77.89	

Female	16	22.86	86	14.19	

Transgender	6	8.57	48	7.92	

					

**Age at diagnosis (years)**					0.0637

13–29	8	11.43	82	13.53	

30–39	22	31.43	244	40.26	

40–49	25	35.71	214	35.31	

50+	15	21.43	66	10.89	

					

**Race/ethnicity**					0.5319

White	27	38.57	248	40.92	

African American	28	40.00	237	39.11	

Latino	10	14.29	99	16.34	

Asian/Pacific Islander/Native American/Other	5	7.14	22	3.63	

					

**Risk group**					0.2825

Men who have sex with men	19	27.14	123	20.30	

Injection drug user	31	44.29	241	39.77	

Men who have sex with men and inject drugs	17	24.29	207	34.16	

Heterosexual/Other	3	4.29	35	5.78	

					

**Insurance status**					0.0695

Public	26	37.14	205	33.83	

Private	3	4.29	5	0.83	

None	40	57.14	383	63.20	

Unknown	1	1.43	13	2.15	

					

**Initial AIDS diagnosis**					0.9964

CD4 count <200 cells/mm^3^	55	78.57	476	78.55	

Opportunistic illness	15	21.43	130	21.45	

					

**CD4 count at diagnosis (mean cells/mm^3^)^‡^**		176		172	0.8368

					

**Ever received highly active antiretroviral therapy**					0.1052

Yes	56	80.00	429	70.79	

No	14	20.00	177	29.21	

					

**Ever received prophylaxis against *Pneumocystis jirovecii *pneumonia**					0.7236

Yes	47	67.14	394	65.02	

No	23	32.86	212	34.98	

					

**Ever received prophylaxis against *Mycobacterium avium *complex**					0.9886

Yes	19	27.14	164	27.06	

No	51	72.86	442	72.94	

* Includes 35 additional cases not originally recorded as homeless at diagnosis but who matched with the housing database and had a move-in date that was later than the AIDS diagnosis date.

†Chi square test for differences in proportions and t test for differences in means.

‡Excludes 23 cases that were missing CD4 results.

Of the 676 persons characterized in Table 4, 652 had complete data and were included in the proportional hazards model. Of these, there were two deaths among persons who received supportive housing, 219 deaths among those who were not housed, and 431 censored cases. After adjusting for potentially confounding variables, obtaining supportive housing was independently associated with an 80% reduction in mortality (RH 0.20; 95% CL 0.05, 0.81; Table 5). Mortality also was significantly lower for African Americans and persons with higher CD4 counts at diagnosis and significantly higher for persons 40 years or older at diagnosis and injection drug users.

**Table 5:** Multivariable predictors of mortality among persons who were homeless at AIDS diagnosis in San Francisco, 1996–2006 (N = 652)*

**Characteristics**	**Adjusted Relative Hazard**	**95% Confidence Limits**
		

**Received supportive housing**		

No	Referent	

Yes	0.20	0.05, 0.81

		

**Gender**		

Male	Referent	

Female	0.78	0.51, 1.19

Transgender	0.77	0.43, 1.38

		

**Age at diagnosis (years)**		

<40	Referent	

≥ 40	1.46	1.10, 1.94

		

**Race/ethnicity**		

White	Referent	

African American	0.73	0.54, 1.00

Latino	0.97	0.64, 1.46

Asian/Pacific Islander/Native American/Other	0.55	0.22, 1.35

		

**Risk group**		

Men who have sex with men	Referent	

Injection drug user	1.87	1.24, 2.82

Men who have sex with men and inject drugs	1.47	0.97, 2.22

Heterosexual/Other	1.45	0.67, 3.13

		

**Initial AIDS diagnosis**		

CD4 count < 200 cells/mm^3^	Referent	

Opportunistic illness	1.31	0.94, 1.82

		

**CD4 count at diagnosis^†^**	0.92	0.86, 0.97

		

**Ever received highly active antiretroviral therapy**		

No	Referent	

Yes	1.08	0.79, 1.47

		

**Ever received prophylaxis against *Pneumocystis jirovecii *pneumonia**		

No	Referent	

Yes	0.82	0.60, 1.11

		

**Ever received prophylaxisagainst *Mycobacterium avium *complex**		

No	Referent	

Yes	1.01	0.75, 1.37

* Excludes cases without follow-up or with missing values.

^† ^Included as a continuous variable using incremental categories of 50 cells.

To assess the possible effect of misclassification due to some housed patients not being initially coded as homeless in the AIDS registry, we repeated the multivariable Cox proportional hazards regression excluding these 35 cases. The RH for receiving housing (0.29) was similar to that found in our main analysis (0.20), although with this smaller sample the 95% CL included one (0.07, 1.20).

It is possible that unmeasured characteristics could explain the 80% reduction in mortality from supportive housing, and we determined the conditions that would be needed for this to occur (see Appendix). Specifically, we considered binary confounders of prevalence from 10% to 90% and correlations with not receiving housing that ranged from 0.1 to 0.5. For each combination, we calculated the strength of the association between the omitted confounder and mortality that would completely explain the reduction. In one scenario, we found that an unmeasured confounder affecting 70% of the population and correlated at 0.5 with not receiving supportive housing would be needed to cause a nine-fold increase in mortality to explain the apparent protective effect of receiving housing.

### Estimating the cost per year of life saved of providing housing

We identified 49 case-control pairs. The restricted mean survival time during the first five years of follow-up was 57.2 months (95% bootstrap CL, 51.7, 60.0) for the housed cases and 42.5 months (95% bootstrap CL, 33.4, 51.2) for the matched controls, resulting in an average gain of 14.7 months. At an approximate cost of $1,000 per month, the average cost of housing per year of life saved was $ 46,800 (95% bootstrap CL, $28,946, $171,746).

## Discussion

Homeless persons with AIDS had significantly worse survival than housed persons, and the provision of housing after AIDS improved survival. These findings have important policy implications given that supportive housing is a feasible and affordable intervention.

Several factors associated with homelessness are likely to have contributed to the poorer survival in this group, including drug use, serious mental illness, inadequate use of health care, and poor adherence to medications. Consistent with prior studies, antiretroviral use was lower among homeless compared to housed AIDS cases [17,18,21]. Although we adjusted our model for initiation of antiretroviral use, we did not have data on compliance with regimens, length of time patients were treated, adverse reactions, or development of resistance. Several studies among HIV-infected homeless persons have documented high rates discontinuation of antiretroviral medications and treatment interruption [17,22]. In addition, HIV-infected homeless persons have fewer ambulatory care visits than infected persons who are housed [20]. These unmeasured factors could bias away from the null our estimate of the adverse effect of homelessness.

Injection drug use was more common among homeless than housed persons and was an independent predictor of mortality in our study as well as in others [32,33]. Injection drug use has been associated with premature mortality, [34,35] due in large part to overdose, suicide, homicide, accidents, and liver disease [34,36,37]. A large proportion of the non-HIV-related deaths among homeless AIDS cases was due to these drug-related causes.

Persons with heterosexually acquired HIV were at increased risk of death. This finding is consistent with some [33,38] but not all [32,39] studies of AIDS survival. Previous studies of persons with HIV/AIDS in San Francisco and nationally have documented that compared with MSM, heterosexuals are more likely to be diagnosed with HIV within 12 months of AIDS suggesting that they are diagnosed with HIV later in the course of their disease, a factor associated with worse survival [38,40].

Persons lacking private health insurance had an increased risk of death. This finding is supported by other studies of HIV-infected persons [24,41] and may reflect differential access to care and/or treatment [42-45]. It is likely that persons with private insurance received medical care by private physicians who may be more available to manage adverse reactions to medications and to facilitate uninterrupted use of antiretroviral therapy than providers in public settings.

Prior studies have shown that housing the homeless, including those with mental illness and substance abuse problems, is feasible [46-48] and can be accomplished for a modest cost [49]. Our study advances the field by demonstrating that supportive housing can decrease mortality. This finding may be due to housed persons being better able to keep medical appointments, store their medications, and adhere to their regimen because housing provides a structure that contributes to a steady routine. The medical and case management services that were provided with the housing may have also contributed to the gains in survival. Indeed, case management has been shown to reduce unmet need and improve use of antiretroviral medications among HIV-infected persons [50,51] and reduce the number of hospital days and emergency department visits among homeless persons with chronic medical illness [48]. Housing may also result in decreased substance use among HIV-infected persons [52].

This study has several limitations. We defined an individual as homeless at diagnosis based upon information in the medical record. It is possible that persons with unstable housing gave an address at the time they were diagnosed that reflected a temporary housing situation, such as staying with a friend or relative, and would have been misclassified as housed. Also, an individual who was stably housed at diagnosis could have become homeless at a later date. Similarly, someone who was homeless at diagnosis could have subsequently received housing. All of these scenarios would have resulted in minimizing the differences in survival between the housed and homeless groups.

Although few AIDS cases are unreported, we investigated the possibility that differences between reported and unreported cases could have biased our results. The AIDS surveillance system is evaluated annually for completeness of reporting. This is done at selected sites where ICD-10 codes that correspond to HIV, AIDS, and HIV-related conditions are used to identify persons who may have HIV. The list is matched against the HIV/AIDS registry to identify persons who may have been missed. The medical records of persons who did not match with the registry are reviewed to identify persons with HIV/AIDS. Any missed cases are reported and the reporting source is listed as coming from the evaluation. We examined characteristics of cases that were initially not reported but later found during annual evaluations of the surveillance system for the years 1996 through 2002. Missed cases were significantly (p < 0.05) more likely to be housed, white, alive at diagnosis, and to have private health insurance (data not shown). Thus, in order for unreported cases to have biased our results, these cases would have to have worse survival than the reported housed cases, a scenario that we believe is unlikely. Missed cases may impact the generalizability of our findings but because AIDS case reporting is so complete, such an effect would be small.

In our analysis we used all cause mortality. We know that homeless persons are more likely to die from non-AIDS-related causes than are housed persons. To assess the impact of using all cause mortality, we excluded persons whose primary cause of death was not HIV/AIDS and conducted another Cox proportional hazards model with this restricted dataset. Our findings were essentially unchanged (RH for homeless 1.19; 95% CL 1.00, 1.42).

As with any nonrandomized comparison of an intervention, the most significant limitation to our analysis is the possibility that persons who were housed were not equivalent to those who remained homeless in unmeasured, or measured but inadequately modeled, characteristics. In particular, if those who received housing were more likely than those who were not housed to survive even if they did not receive housing, the validity of the comparison would be undermined. Several factors argue against this outcome applying to this study. First, the supportive housing program seeks to house people with the greatest medical and/or psychosocial need. Second, the comparisons between housed and not housed persons showed the groups to be remarkably similar. The strongest difference was that the housed group was more than twice as likely to be aged 50 years or more, a strong predictor of worse survival, and corroborating evidence that the program houses persons in greatest need. Persons who were homeless at diagnosis may have received housing subsequently from sources other than the DAH program, which would have caused underestimation of the effect providing supportive housing has on mortality. The results of the sensitivity analysis indicate that it is unlikely that unmeasured confounding could account for our findings.

Although this study used a population-based sample of persons with AIDS, the findings may not be representative of persons with AIDS outside of this geographic region. In San Francisco, HIV is overwhelmingly a disease of MSM. Although this risk group still accounts for the majority of cases nationwide, heterosexual injection drug users and heterosexual partners of injection drug users account for a larger proportion of AIDS cases elsewhere than occurs in San Francisco.

The annual costs per person housed are comparable to those reported from a multisite study of supportive housing for HIV-infected persons [53] and the cost of supportive housing per year of life saved ($46,800) was within the range of approved medical interventions [54,55]. Because of our small sample size, the upper 95% CL exceeded the usual cut-off for cost-effective interventions. Our cost-effectiveness analysis, however, was very conservative in that we considered only the direct cost of the intervention and did not include any of the expected medical care reductions due to providing supportive housing [46]. A large study of supportive housing for the mentally ill in New York City found that 95% of the costs of supportive housing are recouped through savings on hospitalizations and shelter stays [56]. Assuming that only 70% of these costs were recouped, the cost per year of life saved becomes highly cost-effective in our population ($14,040). The cost-effectiveness of housing also would be enhanced if it were quality adjusted, given the gain in quality of life due to being housed versus living on the street.

## Conclusion

This study presents observational data showing a survival benefit from supportive housing. Only a randomized controlled trial could definitively demonstrate that supportive housing reduces AIDS mortality but the follow-up time required for such a study would make it difficult to complete. In the absence of such trial data, we believe that the strengths and plausibility of our findings demonstrate the need to make housing programs a priority for persons with AIDS. Unfortunately, the major federal program for the care of HIV-infected persons, the Ryan White CARE Act, explicitly restricts localities from using funds for long-term housing. Policy makers should recognize that supportive housing can be a cost-effective intervention for reducing mortality in persons with AIDS and provide the mechanisms necessary to ensure that persons in need may receive its benefits.

## Competing interests

The authors declare that they have no competing interests.

## Authors' contributions

SS contributed to the conception and design of the study, data analysis and interpretation. She drafted the manuscript and provided critical review for important intellectual content of the manuscript and participated in all aspects of the data analysis. She had full access to all of the data in the study and takes responsibility for the integrity of the data and the accuracy of the data analysis. LH contributed to the data analysis and interpretation and critical revision of the manuscript. EV contributed to the data analysis and interpretation and critical revision of the manuscript. AV contributed to data analysis and interpretation and review of the manuscript. JB contributed to the acquisition of the data, critical revision of the manuscript, and administrative and technical support and supervision for the housing program. MK conceived the study design, contributed to the data analysis and interpretation, and provided critical review for important intellectual content of the manuscript. All authors read and approved the final manuscript.

## Appendix: Assumptions and formula used in the sensitivity analysis

Suppose that the true RH for the factor of interest, after adjustment for the unmeasured confounder, is actually 1.0, or equivalently the true regression coefficient *β*_1 _is zero. Then *β*_2_, the coefficient or log-RH for the unmeasured confounder, has to be approximately



where *b*_1 _is the confounded estimate of *β*_1_, *f*_1 _and *f*_2 _are the prevalence of the primary predictor and unmeasured confounder respectively, and *r *is the correlation between them.

## Pre-publication history

The pre-publication history for this paper can be accessed here:


